# Temperature Differentially Facilitates Spontaneous but Not Evoked Glutamate Release from Cranial Visceral Primary Afferents

**DOI:** 10.1371/journal.pone.0127764

**Published:** 2015-05-20

**Authors:** Jessica A. Fawley, Mackenzie E. Hofmann, Tally M. Largent-Milnes, Michael C. Andresen

**Affiliations:** Department of Physiology and Pharmacology, Oregon Health and Science University, Portland, Oregon, United States of America; The Research Center of Neurobiology-Neurophysiology of Marseille, FRANCE

## Abstract

Temperature is fundamentally important to all biological functions including synaptic glutamate release. Vagal afferents from the solitary tract (ST) synapse on second order neurons in the nucleus of the solitary tract, and glutamate release at this first central synapse controls autonomic reflex function. Expression of the temperature-sensitive Transient Receptor Potential Vanilloid Type 1 receptor separates ST afferents into C-fibers (TRPV1+) and A-fibers (TRPV1-). Action potential-evoked glutamate release is similar between C- and A-fiber afferents, but TRPV1 expression facilitates a second form of synaptic glutamate release in C-fibers by promoting substantially more spontaneous glutamate release. The influence of temperature on different forms of glutamate release is not well understood. Here we tested how temperature impacts the generation of evoked and spontaneous release of glutamate and its relation to TRPV1 expression. In horizontal brainstem slices of rats, activation of ST primary afferents generated synchronous evoked glutamate release (ST-eEPSCs) at constant latency whose amplitude reflects the probability of evoked glutamate release. The frequency of spontaneous EPSCs in these same neurons measured the probability of spontaneous glutamate release. We measured both forms of glutamate from each neuron during ramp changes in bath temperature of 4–5°C. Spontaneous glutamate release from TRPV1+ closely tracked with these thermal changes indicating changes in the probability of spontaneous glutamate release. In the same neurons, temperature changed axon conduction registered as latency shifts but ST-eEPSC amplitudes were constant and independent of TRPV1 expression. These data indicate that TRPV1-operated glutamate release is independent of action potential-evoked glutamate release in the same neurons. Together, these support the hypothesis that evoked and spontaneous glutamate release originate from two pools of vesicles that are independently modulated and are distinct processes.

## Introduction

Thermodynamics govern all biological processes with substantially different sensitivities for different processes [1]. This holds true for the kinetics of synaptic transmission which are generally accelerated at near-physiological temperatures compared to room temperature [2, 3]. However, many synaptic related studies utilize large temperature changes that are prolonged and often include quite non-physiological temperatures for mammals (e.g. room temperature) [4–6]. Our neurophysiological studies focused on synaptic transmission at rat brainstem neurons of the solitary tract nucleus (NTS) and the temperature-sensitivity of cranial visceral primary afferent transmission compared between afferents that express TRPV1 channels to those that do not [7–11]. Functionally, TRPV1 activation has two actions particularly important for synaptic transmission. As a cation channel, TRPV1 activation directly depolarizes the membrane leading to excitation. However sustained, intense activation of TRPV1 inactivates voltage-dependent channels and suppresses action potential generation [12]. Secondly, the opening of highly Ca^+2^ permeable TRPV1 channels raises intracellular Ca^+2^ levels that increases neurotransmitter release in sensory synaptic terminals [13–15]. While the canonical threshold for gating TRPV1 is ∼43°C in peripheral somatic afferents, physiological temperatures near 37°C may be more relevant at central synapses [7]. At brainstem central primary synapses, normal physiological temperatures activate TRPV1 and increases spontaneous glutamate release [7, 8, 10]. Consequently, small fluctuations in temperature alter the frequency of spontaneous glutamate release at TRPV1 expressing synapses.

Afferents in the NTS serve as a unique system to test the probability of evoked glutamate release for comparison independently from the probability of spontaneous glutamate release. Two distinct afferent phenotypes are differentiated by TRPV1 expression and that corresponds to myelinated (TRPV1-) and unmyelinated (TRPV1+) cranial primary afferent axons [16]. Electrical activation of all solitary tract afferents (ST) evokes monosynaptic excitatory synaptic currents (ST-eEPSCs) while spontaneous events occur from the same afferent during unstimulated periods (i.e. spontaneous EPSC or sEPSCs). In addition to the evoked and spontaneous forms of glutamate release, only TRPV1+ afferents have an extra, TRPV1-operated form of glutamate release. We have recently demonstrated that these forms of release can be independently modulated [11].

A major goal of this work was to test whether temperature changes near the physiological range alter spontaneous and evoked release of glutamate at NTS second order neurons. Surprisingly, the results suggest that axon conduction measured as the ST-eEPSC latency closely and continuously follows temperature whereas the amplitudes of these evoked events were temperature-independent—regardless of TRPV1 expression. In contrast, spontaneous release of glutamate (sEPSC rate) in these same neurons only followed temperature if TRPV1 was expressed in the ST afferent. These thermal data suggest that evoked and spontaneous release arise from distinct presynaptic processes with unique thermal sensitivities and provide further evidence in support of two independent pools of glutamate vesicles that are individually controlled.

## Methods

### Ethical Approval

All animal procedures were approved by the Institutional Animal Care and Use Committee at Oregon Health and Science University (protocol # IS00002752) and conform to the National Institutes of Health guidelines. Twenty-five male Sprague-Dawley rats (150–250 g, Charles Rivers Laboratory) were used. Animals were housed under 12 h light/12 h dark conditions and fed standard pellet chow *ad libitum*. Brains were removed under deep isoflurane anesthesia (5%) and hindbrain slices prepared as previously described [17].

### Brain slices

We removed a wedge of ventral brainstem so that horizontal slices (250 μm) of the hindbrain contained the ST in the same plane as the cell bodies in the caudal NTS (Leica VT-1000S vibrating microtome, Leica Microsystems Inc., Bannockburn, IL and sapphire blade, Delaware Diamond Knives, Wilmington, DE). Slices were submerged in a perfusion chamber in an artificial cerebrospinal fluid (ACSF) composed of (mM): 125 NaCl, 3 KCl, 1.2 KH_2_PO_4_, 1.2 MgSO_4_, 25 NaHCO_3_, 10 glucose, and 2 CaCl_2_, that was bubbled with 95% O_2_ / 5% CO_2_ (pH 7.4). The chamber was continuously perfused (1–2 ml/min) with ACSF held within 1˚C using an in-line heating system (Cell MicroControls, Norfolk, VA).

### Temperature controlled bath perfusion system

We maintained brain slices by continuous perfusion of the recording chamber with artificial cerebrospinal fluid (ACSF). ACSF was delivered through a roller pump (Instech P720) from a solution reservoir selected via a solenoid pinch-valve assembly and inline pre-heater (HPRE2HF and TC^2^ controller (Cell MicroControls). To accomplish dynamic bath temperature changes, external temperature commands generated by a programmable stimulator (Master-8/9, A.M.P.I., Jerusalem, Israel) produced slow changes in chamber temperature. The command protocols consisted of a series of short duration voltage command levels that resulted in ramps of bath temperature at controlled rates and times. Bath temperature was continuously measured by a thermistor placed in the bath solution downstream from the slice.

### Patch-clamp recording and afferent activation

Patch pipettes were pulled from borosilicate glass (NTS: 2.0–3.6 MΩ) and filled with (mM): 6 NaCl, 4 NaOH,130 K-Gluconate, 11 EGTA, 1 CaCl_2_, 2 MgCl_2_, 10 HEPES; 2 Na_2_ ATP, 0.2 Na_2_ GTP; pH adjusted to 7.3–7.32. NTS neurons were targeted for recording by visualization using infrared differential interference contrast optics (Zeiss Axioskop FS2, Thornwood, NJ) in a region within ± 250 μm rostral-caudal to the caudal end of the 4^th^ ventricle and medial to the ST. Neurons were voltage clamped and held at -60 mV (Multiclamp 700B, Axon Instruments, Union City, CA) and synaptic currents sampled at 20 kHz and filtered at 10 kHz using pClamp 9.2 software (Axon Instruments). Liquid junction potentials were corrected in current clamp experiments. Electrical activation of the afferent axons was accomplished by placing a concentric bipolar stimulating electrode (200 μm outer tip diameter; Frederick Haer Co., Bowdoinham, ME) on the ST >1 mm from the recorded neuron. Stimulation used constant current shock protocols programmed in the Master-8 (100 μs duration shocks delivered most commonly in sets of 5 at 50 Hz repeated every 6 or 10s) and tested with graded shock intensity to recruit minimal ST-eEPSCs and avoid compound events as much as possible. TRPV1 classification was assessed functionally using 100 nM capsaicin [7, 11, 16].

## Results

### Assessing distinct stages of presynaptic transmission

**Fig 1:**
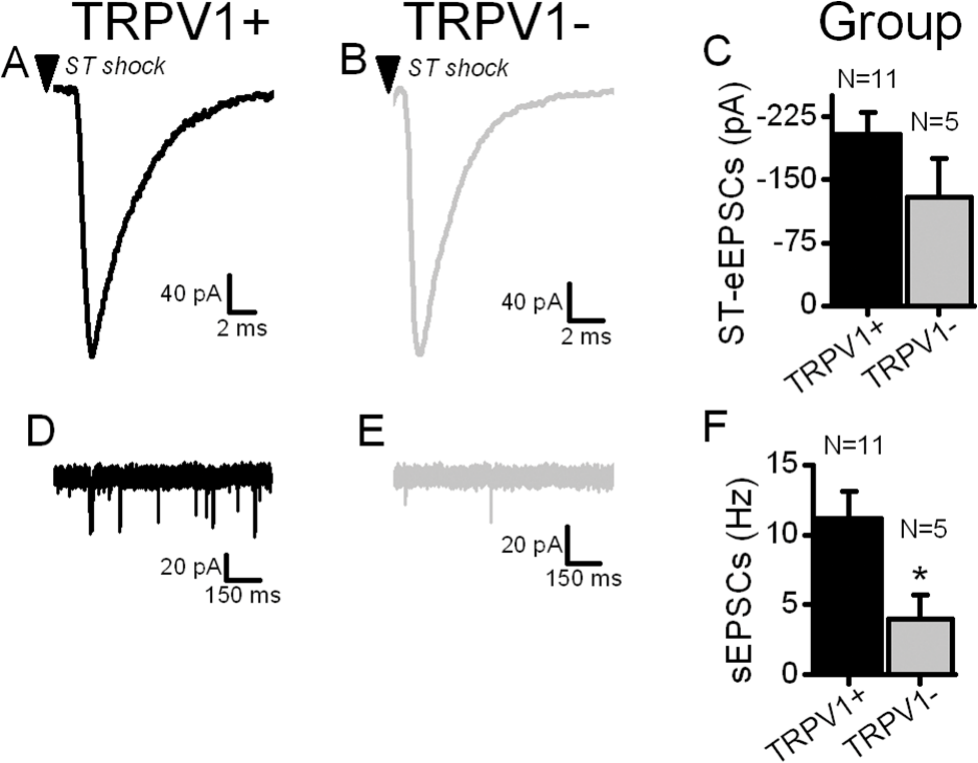
Two phenotypic classes of second order solitary tract (ST) neurons have similar ST-evoked excitatory synaptic current (EPSC) responses but very different spontaneous EPSC rates between TRPV1+ and TRPV1- ST afferents. Shocks to the ST (arrowheads) evoked time-invariant glutamate release from TRPV1+ (A) and TRPV1- (B) types of neurons. Across neurons (C), evoked glutamate release was indistinguishable between afferent types (p = 0.2, t-test). Spontaneous EPSC rates (i.e. sEPSCs) in TRPV1+ neurons (D) were higher than TRPV1- neurons (E).Original traces are from the same neurons; A and D representing a single TRPV1+ neuron while B and D are from the same TRPV1- neuron. Across neurons (F), the rate of sEPSCs is much higher (p = 0.04, t-test) than from TRPV1- afferents. The results suggest that TRPV1+ afferents have a more active spontaneous glutamate release process than TRPV1-. Exposure to 100 nM capsaicin tested whether neurons were TRPV1+ or TRPV1- (not shown).

Action potentials travel along the primary afferent axon until reaching the synaptic terminal where they depolarize the terminal and trigger voltage-activated calcium channels (VACCs) to release glutamate [18]. ST-eEPSC latency largely reflects the conduction process independent from release [19]. Two phenotypes of ST afferents (Fig 1) are distinguished by whether or not they respond to TRPV1 activation by agonists and correspond to unmyelinated TRPV1+ or myelinated TRPV1- ST axons, respectively [7, 11, 16]. The process of monosynaptic evoked transmission is indistinguishable between myelinated and unmyelinated ST afferents (Fig 1A–1C) [20] and its timing is so reproducible that the variation in arrival times (jitter, SD of latency) can be as low as 30–40 μs. However, recent evidence indicates that unmyelinated cranial visceral afferents possess a second calcium entry pathway through TRPV1 which is responsible for spontaneous release from a pool of glutamate vesicles separate from those responsible for evoked release [21, 22]. Note that ST primary afferents with myelinated axons display much lower spontaneous synaptic activity (sEPSCs) than from unmyelinated ST afferents (Fig 1D and 1E). In the present studies, we use small changes in bath temperature to simultaneously assess the thermal dependence of the conduction and release processes of action potential evoked release compared to the spontaneous synaptic release process.

### Temperature-evoked changes of spontaneous EPSC frequencies are limited to TRPV1+ ST afferents

**Fig 2:**
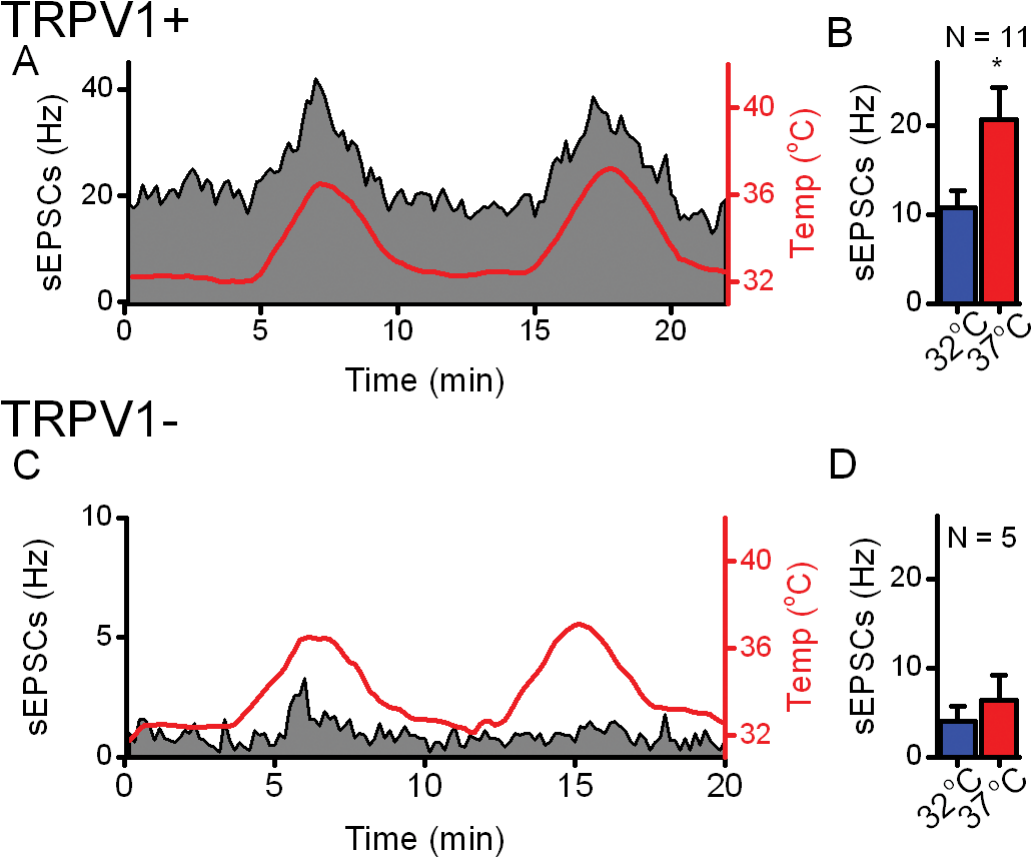
The rate of sEPSCs in TRPV1+ neurons closely track with temperature but not in TRPV1- neurons. We applied two ramps (0.1 V every 20 s) to generate changes in bath temperature (red) from 32° to 37°C and back to 32°C while sEPSCs were continuously recorded from second order NTS neurons. (A) In a representative TRPV1+ neuron, sEPSC frequency (10 s bins black/filled grey) tracked with bath temperature (10 s bins). (B) Across neurons, the average frequency of sEPSCs more than doubles with a 5°C increase in temperature (p < 0.01, Two Way RM ANOVA). (C) sEPSCs from this TRPV1- neuron do not track with temperature. (D) In a representative TRPV1- neuron, TRPV1 sEPSCs failed to track with temperature. (D) Across TRPV1- neurons, sEPSCs frequency was unchanged (p = 0.3, Two Way RM ANOVA).

The spontaneous release process is distinctly different between neurons receiving TRPV1+ and TRPV1- ST afferents [7]. The rate of sEPSCs was ten-fold higher in TRPV1+ compared to TRPV1- ST afferents and elevation of temperatures increased the rate of sEPSCs only in ST afferents expressing TRPV1 [8]. The spontaneous release of glutamate onto NTS neurons requires neither Na^+^ nor Ca^++^ channel activity [9]. To examine the dynamics of thermal actions on the spontaneous release process, brief temperature ramps were tested (Fig 2). Note that the rate of sEPSC frequency was generally much higher in TRPV1+ neurons and that ramps of increasing temperature reproducibly increased the sEPSC rate (Fig 2A). However, in neurons that received TRPV1- ST afferents, the sEPSC rate was much lower and failed to track with bath temperature changes (Fig 2C). Across neurons, the average basal sEPSC rate substantially increased (222 ± 20%) as temperature increased from 32°C to 37°C (Fig 2B). In contrast, the sEPSC rates of NTS neurons with TRPV1- afferents (n = 6) were not significantly changed (159 ± 15%) by these temperature shifts (Fig 2D). Afferents that displayed thermal sensitivity also responded with increased sEPSC rate to the TRPV1 agonist capsaicin (100 nM CAP, data not shown) conclusively identifying TRPV1 whereas thermally-insensitive neurons (i.e. TRPV1-) did not respond to CAP. Therefore thermal tests effectively served as a drug-free surrogate for TRPV1 agonist responses.

### Warming progressively decreases latencies but not amplitudes of ST-evoked EPSCs regardless of TRPV1 expression

**Fig 3:**
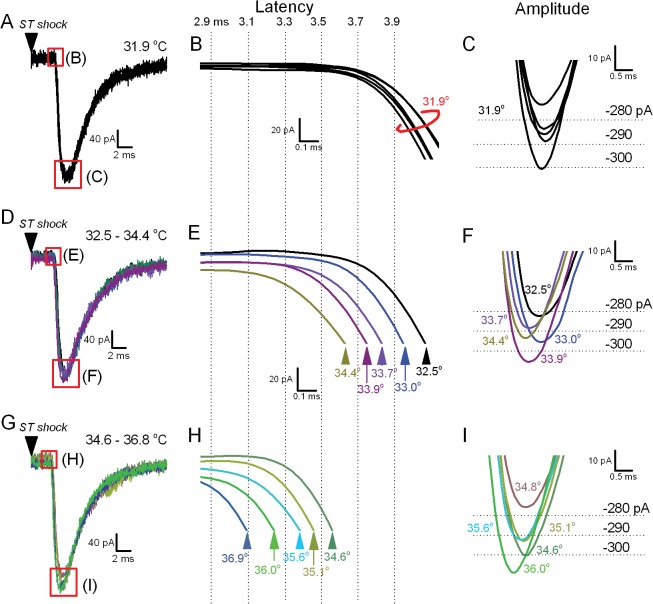
Increases in bath temperature progressively shortened the evoked ST-EPSC latency but event amplitudes were constant. In a single, representative TRPV1+ NTS neuron, latencies (black, A, B) were quite consistent over 5 representative trials of ST shocks with temperature held at a steady 31.9°C. B is expanded to show just the initial inward current of the ST-eEPSC. Note that the amplitudes (A, expanded in C) normally vary somewhat from trial to trial despite fixed conditions. (D-I) As temperature is increased, ST-eEPSCs arrive progressively earlier (unique colors for each temperature (E-F) and (H-I)). Note that event amplitudes are similarly variable, not ordered by temperature and have similar averages (p = 0.1, One way ANOVA). Traces were low-pass filtered for clarity (Gaussian). Dotted lines represent the indicated times (in ms) elapsed from the solitary tract shock across all 3 middle panels (B, E, H).

The latency from ST shock to the onset of the ST-eEPSC primarily reflects the axonal conduction process since it measures the time it takes the action potential to travel from the location of the ST shock to the synaptic terminal and trigger neurotransmitter release—a remarkably consistent process at fixed temperatures. The magnitude of the evoked glutamate release process is measured by the ST-eEPSC peak amplitude and depends directly on VACC-mediated calcium entry [7, 23, 24]. Here we compare the effects of temperature on both the timing and the magnitude of the evoked EPSC. Within single recordings, the ST-eEPSCs were stable in timing and amplitude when temperature was held constant (Fig 3A and 3B). Warming from 32 to 37°C shifted the latency gradually to shorter arrival times indicating faster conduction (Fig 3D, 3E, 3G and 3H). The shifts in ST-eEPSC arrival times were evident even with temperature changes of less than 1°C (Fig 3B, 3E and 3H). In contrast, the amplitudes of these same ST-eEPSC events showed no relationship to the same temperature shifts (Fig 3C, 3F and 3I). The data suggest that eEPSC conduction was highly temperature dependent but glutamate release was unaltered.

**Fig 4:**
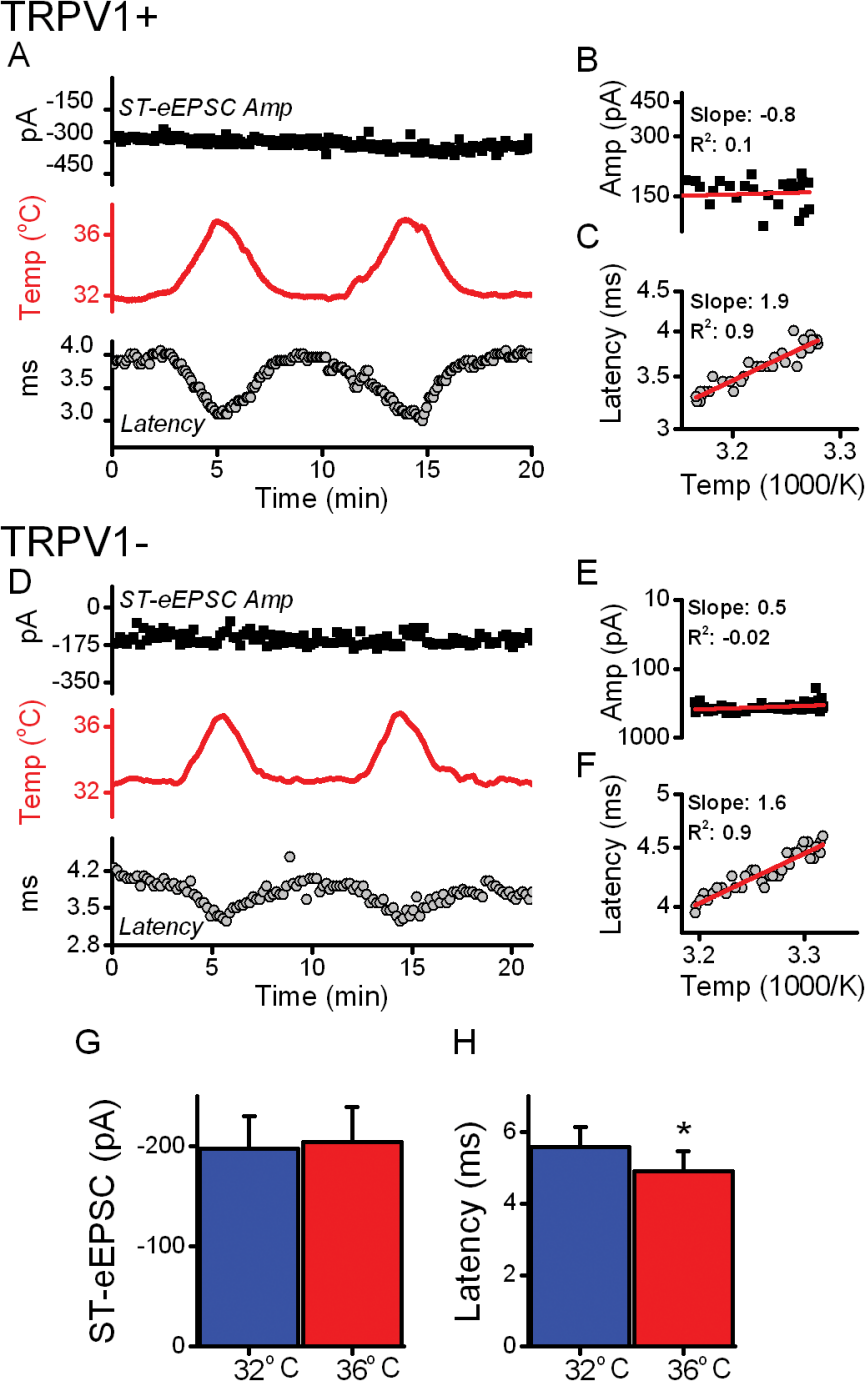
Temperature rapidly and reversibly modified arrival times for ST evoked EPSCs similarly in both TRPV1+ and TRPV1- afferent NTS neurons. Diary plots (A, D) show the effect of temperature on both the latency and amplitude of ST-eEPSCs. Temperature (red, middle panels) had an inverse relationship with latency (grey circles) but event amplitudes (black squares) remained constant. Arrhenius relations for the same data plot the log amplitude (B, E) or latency (C, F) against the temperature (1000/T(°K)). For latency, this relation was well fit by linear regression with positive slopes (C, F), a measure of the thermal sensitivity. However, fits to the amplitude Arrhenius plots (B, E) had near zero slopes. These patterns were not different between TRPV1+ (A-C) and TRPV1- (D-F) afferents. Across neurons (TRVP1+, n = 6; TRPV1-, n = 3), the latency decreased by an average of 13 ± 2% (p < 0.01, Two Way RM ANOVA) similarly across afferent phenotypes (i.e. TRPV1+ vs. TRPV1-; p = 0.2, Two Way RM ANOVA). Despite this, amplitudes (103 ± 2%) were unchanged by the same temperature shift (p = 0.1, Two Way RM ANOVA).

The close thermal tracking of ST-eEPSC latency but not amplitude was even more apparent in diary plots of values across time during thermal challenges. Latency decreased and then increased in close relation to the changes in bath temperature (Fig 4). Such reversible thermal behavior was remarkably similar whether the neuron was connected to TRPV1+ or TRPV1- ST afferents (Fig 4). Note that amplitudes of ST-eEPSCs varied little in these same representative neurons. Arrhenius plots of these values [25] from these individual neurons had significant, positive slopes for latency but not for amplitudes in both TRPV1+ (Fig 4B and 4C) and TRPV1- (Fig 4E and 4F). Temperature significantly shifted the average latency of ST-eEPSCs by 700 ± 40 μs (Fig 4H) from both afferent types. However, the average amplitudes of these same ST-eEPSCs were unchanged by temperature (Fig 4G). These findings suggest that the axonal conduction aspect of afferent evoked transmission rapidly, reversibly and reliably followed temperature changes regardless of TRPV1 expression but that the final stage of the evoked glutamate release process was unaltered across this near physiological temperature range.

### Temperature increases action potential firing

**Fig 5:**
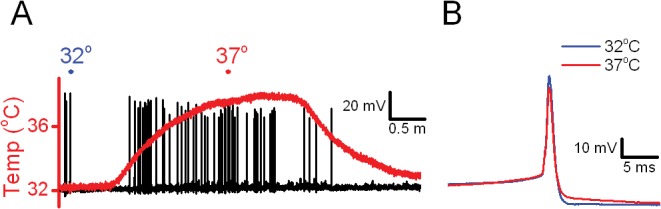
In TRPV1+ NTS neurons, temperature increased the rate of action potential firing. (A) In this current clamp recording trace, warming the bath temperature (red) near 37°C briskly increased the rate of action potentials. The shapes of the action potentials changed little from 32°C (blue) to 37°C (red) in expanded traces (B). Resting membrane potential ~ 57 mV at 32°C.

Normal ambient temperatures in the intact brain are generally near 37°C. Our synaptic work suggests that warming to near 37°C greatly increases stochastic release from afferent glutamatergic terminals on second order brainstem neurons. To test whether this sEPSC barrage generated action potentials, we recorded from cells in current clamp. Action potential “spontaneous” activity increased substantially when temperature was increased from 32° to 37°C (Fig 5). Note that in this range of near physiological temperatures, the shapes of the action potential and the membrane potentials at which the action potential was triggered were quite similar (Fig 5B). Such changes in action potentials indicate that even locally generated TRPV1-operated release will be broadcast beyond the second order neurons effectively generating a stochastic signal from ambient temperature driven TRPV1 gating.

## Discussion

Temperature fundamentally influences the biophysics of excitability, ionic distributions and ion channel function in neurons and other excitable cells [26]. Our present studies focused on synaptic glutamate transmission and compared electrical shock-evoked EPSCs to spontaneous EPSCs. We tested both ST-eEPSCs and sEPSCs responses in the same single neurons and used small continuous changes in temperatures near the physiological range (32 and 36°C) to test the continuity of action across multiple key aspects of excitation-transmission. Our chief findings were that: 1) warming progressively decreased latency but did not alter amplitudes of ST-evoked EPSC regardless of TRPV1 expression. 2) Temperature modulated the frequency of stochastic sEPSCs only in TRPV1+ neurons. Thus, temperature did not affect the probability of release from the synchronously evoked glutamate pool in either TRPV1+ or TRPV1- afferents but did selectively alter spontaneous glutamate release only in TRPV1+ phenotypes. Together, these data provide important evidence that evoked and spontaneous release arise from different pools of glutamate vesicles.

Early physiological studies of peripheral nerve trunks capitalized on large reductions in temperature to block nerve conduction and distinguish axon caliber and type [27]. Small temperature changes near 37°C continuously altered conduction velocity in peripheral nerves [28] and quantitative investigations found decreases in conduction velocity from 37°C to 17°C that were proportionally identical for myelinated and unmyelinated peripheral axons [29]. This is consistent with our finding that the latency of evoked EPSCs in NTS neurons rapidly and reversibly shifted to shorter intervals on warming from 32 to 37°C to closely track temperature, regardless of afferent type. Changes in conduction are generally attributed to Na^+^ channels, which increase in the range of 25–40°C [30], while changes to presynaptic calcium channels do not occur in this near-physiological range [2]. The lack of amplitude changes in our observations over the same temperature range therefore suggests that calcium entry remained relatively constant but there was a higher thermal sensitivity of axon conduction in the evoked transmission process.

The actions of temperature on ST synaptic transmission points to key changes in latency but not the amplitude of the evoked EPSCs, similar to the actions of the local anesthetic QX-314 on these NTS afferents [19]. QX314 is well known to target Na^+^ channel function [31]. Consistent with the thermal profile of ST-eEPSCs found in the present studies, QX-314 had no effect on spontaneous EPSCs in NTS neurons [19]. It is inviting to speculate from the two studies that temperature primarily targets conduction by acting on the Na^+^ channel contribution to the conduction step of ST transmission while thermally-sensitive spontaneous glutamate rates do not depend on Na^+^ channel function but rather TRPV1. The selective thermal sensitivity of TRPV1-operated release might indicate that this mode of glutamate release can be independently modulated (e.g., G-protein coupled receptors), which is supported by recent evidence of the sensitivity to endocannabinoids [11].

While seminal work identified spontaneous miniature quantal release as the building blocks of full scale evoked synaptic events [32–35], recent work suggests that spontaneous release in some neurons can represent an independent signaling mechanism [36–39]. Likewise, evidence in NTS neurons suggests that separate glutamate vesicle pools exist for action potential-evoked transmission and spontaneous events with different mechanisms governing release [7, 11, 40]. These two vesicle pools in ST afferents were independently and oppositely modulated by CB1 and/or TRPV1 activation in single neurons [11]—further evidence of independent release determinants for separate processes that are both regulated by calcium entry.

The present studies examined the influence of temperature on the two hypothesized mechanisms: one regulating the readily releasable pool of glutamate vesicles subject to evoked release and the other regulating a separate, TRPV1-operated pool limited to unmyelinated afferent endings. In our experiments, ST shocks triggered similar evoked release regardless of TRPV1 expression. Temperature (32°–37°C) did not change the mean amplitudes of these evoked events indicating that this fundamental process is unaltered across this temperature range. In contrast, the frequency of spontaneous release (an index often interpreted as reflecting the probability of release) was directly and steeply dependent on temperature over this same range. The spontaneous release process from the TRPV1-operated pool appears to be fundamentally simpler and relies on calcium entry through TRPV1, a sequence that requires neither VACCs nor action potentials [9, 16]. Spontaneous release of glutamate in TRPV1+ NTS neurons appears to be triggered by thermal gating of TRPV1 to accomplish the calcium entry responsible for the high rates of “spontaneous” EPSCs, and this process is greatly attenuated by sub-physiological cooling to 30°C [8]. The current results indicate that this gating of TRPV1 is a rapid and readily reversible process. These kinetic characteristics make it unlikely that generation of an endovanilloid can be responsible for such rapid and reversible responses to temperature but likely reflects increasing open probability of TRPV1.

The relative independence of TRPV1-operated glutamate release from evoked EPSCs is surprising and suggests that the calcium responsible for mediating the two forms of release are sufficiently separated within the terminals so as not to directly influence the release of both pools [41]. Rapid activation of the action potential-evoked pool results in robust frequency-dependent depletion and depressed ST-eEPSC amplitudes (see Fig 2 of [7]) without altering sEPSC amplitudes. The spatial domains occupied by the TRPV1-releasable pool and the evoked pool are not distinct and respond similarly to intracellular calcium buffering of EGTA and BAPTA [7]. However, terminal nano- and microdomains for calcium [41] could well be responsible for the segregated and discrete responses of the two pools even within up to a 2 μm afferent ST terminal [42]. Future studies will aim to investigate the mechanisms governing the two forms of glutamate release.

The present studies supplement the evidence that TRPV1-expressing afferent synaptic terminals possess a second pool of vesicles that operates independently from the readily releasable pool activated by action potentials. The differences in thermal changes in the kinetics of EPSC release mechanisms between ST-eEPSCs and sEPSCs reinforce the pharmacological evidence for independent modulation of the two pools. The presence of such an independent brainstem signaling mechanism may set a tonic level of overall activity within unmyelinated pathways even in the absence of peripheral primary afferent activity.
